# Disordered Near-Field Metasurfaces for On-Chip Wavelength Demultiplexing

**DOI:** 10.1021/acs.nanolett.6c03564

**Published:** 2026-10-02

**Authors:** L. Schmid, J. Schwab, F. Kadriu, R. Horvat, C. Li, S. A. Maier, H. Giessen, H. Ren, M. Hentschel

**Affiliations:** † 4th Physics Institute and Research Center SCoPE, 9149 University of Stuttgart, Pfaffenwaldring 57, 70569 Stuttgart, Germany; ‡ School of Physics and Astronomy, 2541 Monash University, Clayton, Victoria 3800, Australia; § Department of Physics, Imperial College London, London SW7 2AZ, United Kingdom

**Keywords:** Near-field, Disorder, Metasurface, Imaging, Demultiplexing, SPPs

## Abstract

Plasmonic and dielectric building blocks shape the flow of light at boundaries and interfaces. While metasurfaces that consist of plasmonic or dielectric nanophotonic elements have established a framework for shaping the far field, control over near fields remains largely unexplored. Here, we introduce disordered metasurfaces for near-field light shaping. Near-field distributions are created by engineering the interference of surface plasmons launched from scatterers on metallic surfaces. We demonstrate near-field metasurfaces to direct, focus, and demultiplex light. These structures use a strip-off process, resulting in ultrasmooth metallic surfaces suitable for efficient surface plasmon propagation. An optical imaging setup enables retrieval of the local near-field by mapping it into the far-field. Our concept can be generalized to arbitrary near-field distributions, permitting nanoscale routing and sorting of light based on polarization and orbital angular momentum, as well as coupling schemes to emitters and nanoscale systems in ultracompact integrated photonic receivers and demultiplexers.

## Graphical abstract

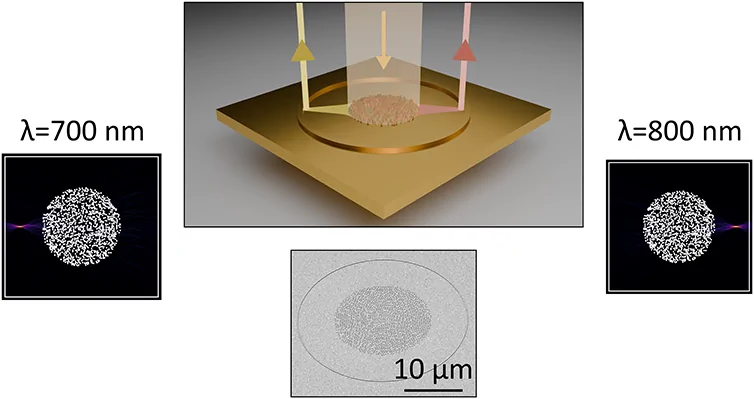

The ability to control and manipulate light at the nanoscale is a central objective in modern nanophotonics, driven by the demand for increasingly compact optical communication,^1–3^ sensing,^4–7^ and information-processing technologies.^8^ In recent years, plasmonic and dielectric nanophotonic structures have enabled unprecedented control over electromagnetic waves at subwavelength dimensions through engineered scattering at surfaces and interfaces. In particular, metasurfaces have emerged as powerful platforms for tailoring the phase, amplitude, and polarization of light with ultrathin optical elements.^9,10^ These concepts have enabled functionalities such as beam steering,^11^ holography,^12,13^ and wavefront shaping in the far field.^14–17^ Such approaches highlight the enormous flexibility of metasurface engineering and illustrate how nanoscale structuring can replace bulky optical components with compact planar architectures. Despite these achievements, metasurface concepts have only recently begun to be explored for the investigation and generation of structured optical near-field distributions, including vortices,^18^ and topological vector textures,^19,20^ in surface waves, especially in surface plasmon polaritons (SPPs). The ability to guide and manipulate these waves at nanoscale dimensions is also applied for ultracompact photonic circuitry and integrated optical devices.^21,22^ Dielectric platforms make use of inverse design approaches in between waveguides.^23,24^ Previous studies demonstrated that plasmon propagation can be selectively controlled through engineered nanostructures and polarization-sensitive couplers, enabling directional launching and routing of SPPs on metallic surfaces.^25,26^ Wavelength-selective devices have been realized based on gratings, concentric grooves,^27^ and dielectric loaded plasmonic waveguides.^28^ The observation is carried out using scanning near-field optical microscopy (SNOM),^29^ as well as leakage radiation in transmission. Building on these developments, we introduce disordered metasurfaces designed specifically for near-field light shaping in combination with an all-optical read-out in reflection. Instead of engineering far-field diffraction patterns, our approach tailors the interference of individually launched surface plasmons through the spatial arrangement of nanoscale scatterers on ultrasmooth metallic films.^30,31^ By controlling the collective surface plasmon response, desired near-field intensity distributions can be generated with high flexibility. Using this concept, we demonstrate metasurfaces capable of directing, focusing, and demultiplexing incident light in the plasmonic near-field with ultracompact footprints.

More specifically, we present wavelength- dependent spatial separation of up to four different wavelengths through engineered interference of SPPs on a smooth gold film. The working principle is illustrated using the four-wavelength design in Figure 1a. Multiple incident wavelengths impinge on the circular area of scattering elements. The plasmons are launched by individual scatterers, which are located such that the generated plasmon waves form wavelength-dependent focal positions. At these positions, the surface plasmons are scattered out of the plane by a scattering ring to a microscope objective (see Figure 2), revealing information about the local near-field intensity.^26,32,33^ Using this approach, we demonstrate three individual devices for the demultiplexing of two, three, and four different wavelengths, respectively. The devices are manufactured using a strip-off process from lithographically defined molds, yielding ultrasmooth metallic surfaces that provide an ideal platform for SPP propagation while simultaneously enabling precise metasurface patterning within the same fabrication process.

**Figure 1:**
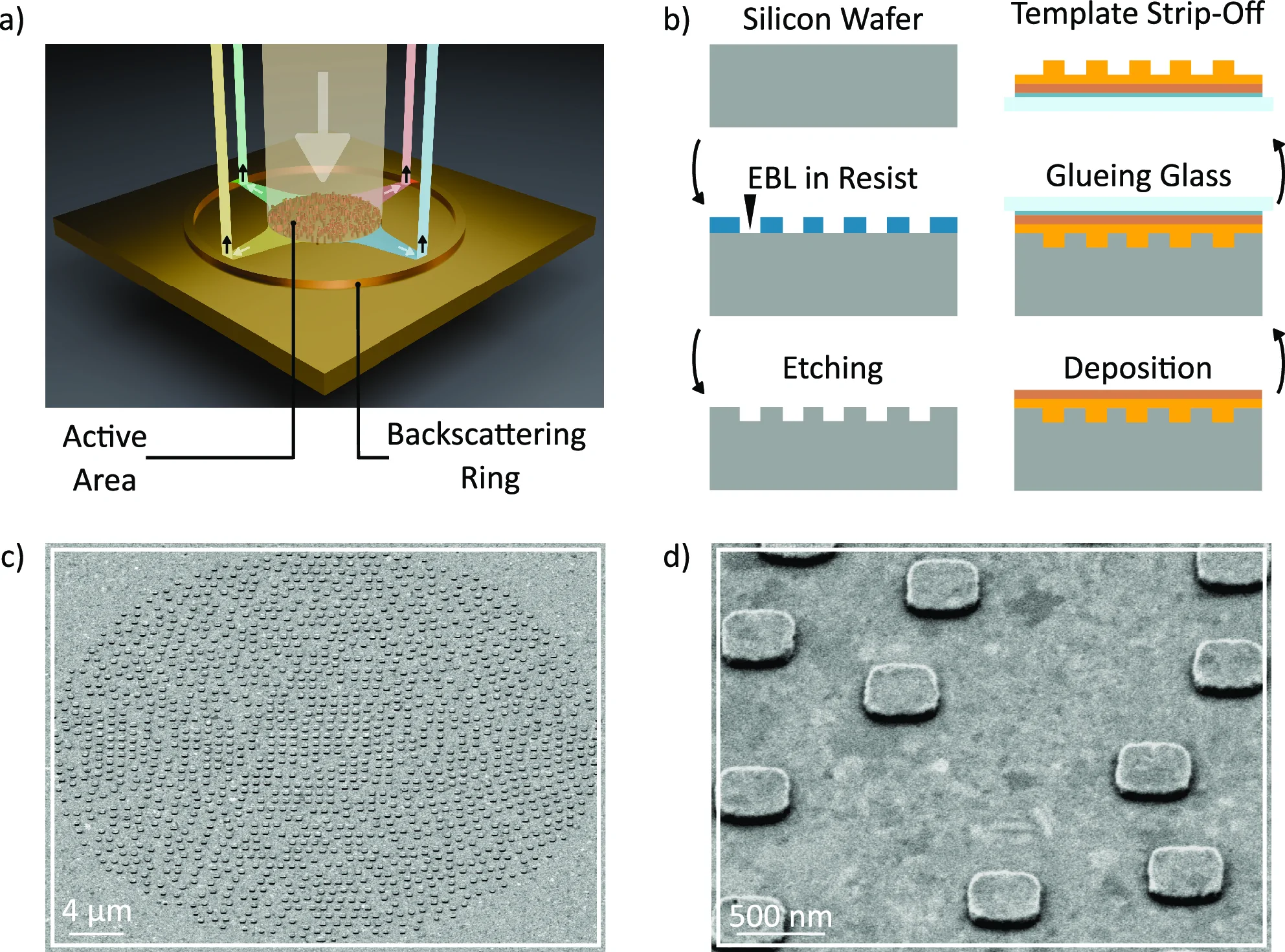
Working principle and fabrication scheme of a disordered near-field metasurface. (a) Schematic illustration of a wavelength demultiplexing device. (b) Fabrication process for ultrasmooth gold surfaces. A polished silicon substrate is subjected to dry etching using an electron-beam (e-beam) lithographically defined mask to generate the desired binary surface topography. Following resist removal, a gold layer is deposited to replicate the patterned silicon structure. Subsequently, a glass substrate is glued to the gold film for template-stripping. (c) SEM image of the entire area containing the individual scatterers, corresponding to the region schematically illuminated in (a). (d) A zoom-in of the individual scatterers fabricated directly in gold.

**Figure 2:**
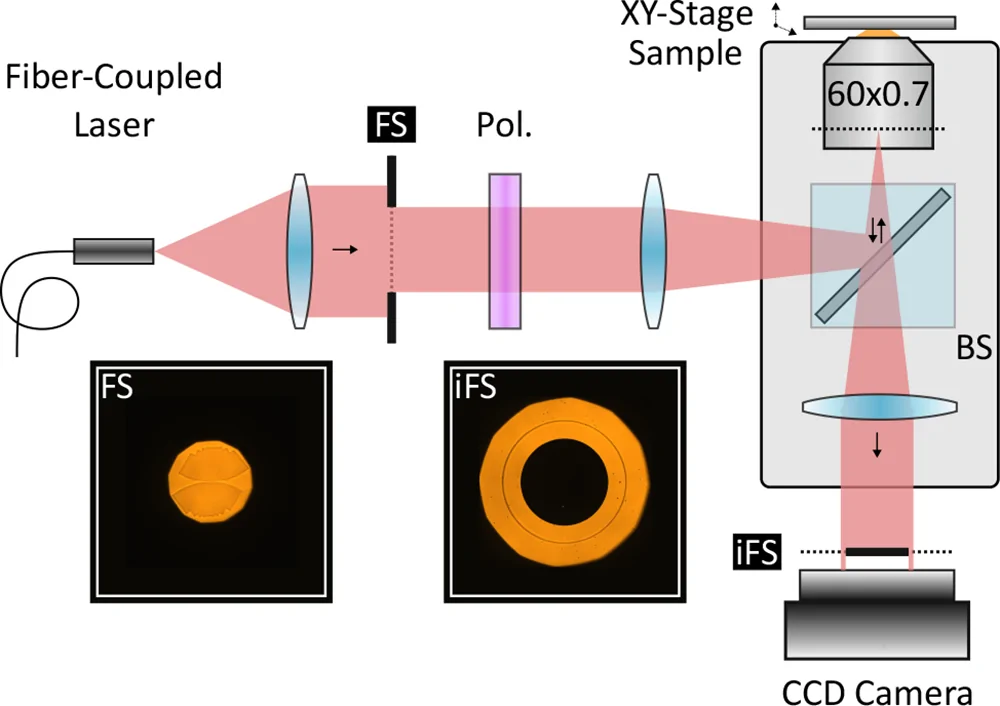
Imaging setup to measure the surface plasmon intensity along the backscattering ring. A fiber-coupled laser source is collimated, and the beam diameter is spatially restricted to match the active area of the nanostructures, as illustrated in the microscope image with the closed field stop (FS). After passing through polarization optics, the beam is focused into the back focal plane of the objective, resulting in an approximately planar wavefront illumination of the sample. The back-reflected light travels via a beam splitter and is imaged onto a CCD camera. Specular reflections are blocked out using an inverse field stop (iFS), as illustrated in the corresponding microscope image.

The fabrication process of our plasmonic device is illustrated in Figure 1b. It starts with a polished silicon wafer exhibiting intrinsically low surface roughness. On top of the wafer, an etching mask (positive-tone electron-beam resist AR-P 6200.13 400 nm, Allresist) is patterned with e-beam lithography (EBL), defining the structures of the individual scatterers as well as the backscattering ring geometry. The resist mask is then transferred into the silicon by 50 nm dry-etching. Subsequently, the resist is removed, and a 100 nm thick gold layer is deposited, followed by an additional 400 nm thick copper layer for stabilization during the lift-off process. Gold was selected owing to its chemical stability and resistance to oxidation, thereby ensuring stable SPP propagation over time. A glass slide is glued onto the deposited copper film. As there is almost no adhesion between the gold and the silicon layer, the glass substrate can be stripped off the silicon wafer once the glue is dry and the glass substrate is removed with the glued gold on top. The gold is extremely smooth, as previously shown for silver by Lassaline et al.,^34^ and directly contains the scattering elements on it. A scanning electron microscope (SEM) image of the scattering elements on gold is depicted in Figure 1c, and a zoom-in on a few individual scatterers is displayed in Figure 1d. These shallow islands demonstrate a distinctive approach for tailoring SPP propagation, highlighting their potential as an effective platform for the control and manipulation of surface waves and providing new opportunities for wave engineering applications. Aside from the individual scatterers, a camouflage-like pattern appears on the gold, which is caused by the growth of individual gold islands. Crystallites exhibit orientation-dependent work functions and electron emission probabilities. This crystallographic anisotropy gives rise to the observed bright–dark contrast, as different crystal orientations generate varying secondary electron yields. Additionally, the presence of the 400 nm copper layer does not influence surface plasmon propagation, since the electromagnetic skin depth is only on the order of 20 to 30 nm.^35,36^

For the observation of the local near-field intensity we introduce a simple optical setup illustrated in Figure 2, thereby circumventing some of the inherent complexities of SNOM and photoemission electron microscopy (PEEM). Typical techniques to measure the near-field of surface plasmon polaritons, which provide a deep insight into the material and in addition to the amplitude, also allow measurement of the SPP phase.^20,37–39^ A fiber-coupled laser (NKT SuperK extreme, filtered by an Acousto-Optic tunable filter NKT SuperK Select+) is used as a light source, providing multiple wavelengths simultaneously (500 nm–900 nm). The divergent beam is collimated and then restricted in diameter by a field stop (FS) to illuminate only the active area, as illustrated in the left panel of Figure 2. After the FS, the required polarization optics are inserted, depending on the metasurface. In case of the two-wavelength demultiplexing device, a half-waveplate is used to rotate the linearly polarized light. For three and four-wavelength demultiplexing, an additional quarter-waveplate is inserted to generate right-handed circular polarization (RCP). After passing a beamsplitter (BS), the polarized beam is focused to the center of the backfocal plane of the objective for an approximately uniform plane-wave illumination on the sample. The part of the plane-wave, which does not excite surface plasmons, results in specular reflections. As the surface plasmons are scattered out of plane at the backscattering ring, both the specular reflection and the scattered SPPs travel via the BS and a collimation lens onto a CCD camera. We insert an inverse field stop (iFS) in a second image plane to image only the surface plasmons and block the specular reflection. The iFS consists of a filled black circle with a diameter of 3000 μm and is fabricated by 3D-printing of the highly absorptive photoresist IP-Superblack.^40,41^ A microscope image of the iFS is illustrated in Figure 2. The highly absorptive aperture almost completely suppresses the specular reflections on the CCD images, resulting in a high contrast of the backscattered surface plasmons.

Next, we outline the design principle of the metasurfaces based on the previously described fabrication scheme. To maximize functional flexibility, the metasurfaces are composed of individual scatterers arranged using an inverse design approach, illustrated in Figure 3. The disordered near-field metasurface depicted in Figure 3 performs wavelength-dependent directional coupling of incident light into SPPs, directing the two wavelength components into opposite propagation directions and focusing them into separate in-plane focal spots. Although the device directs SPPs toward opposing output ports, potentially giving rise to crosstalk, this effect is sufficiently weak to remain negligible in the simulations as well as in the experiment. The design approach considers the time-reversed scenario, calculating the propagation of SPPs inward from the focal positions at the backscattering ring toward the area of the metasurface as shown in Figure 3a. To achieve this, cylindrical Huygens waves are launched at these focal positions. The excitation of these waves is calculated based on the intended incident linear polarization and the corresponding target operational wavelengths (700 and 800 nm). The scattered waves propagate into the central area and form an interference pattern. Scatterer coordinates are then stochastically assigned to locations exhibiting similar phase (ϕ_0_ ± π/5), while preventing an overlap of the scatterers. This produces the engineered-disordered spatial arrangement illustrated in Figure 3d. The functionality of the resulting structure is confirmed using the forward simulations in Figure 3c and e. By launching a Huygens wave at each scatterer position, their total field demonstrates the intended behavior: the waves interfere constructively to produce a focal spot on the left for an incident wavelength at 700 nm, and on the right at 800 nm.

**Figure 3:**
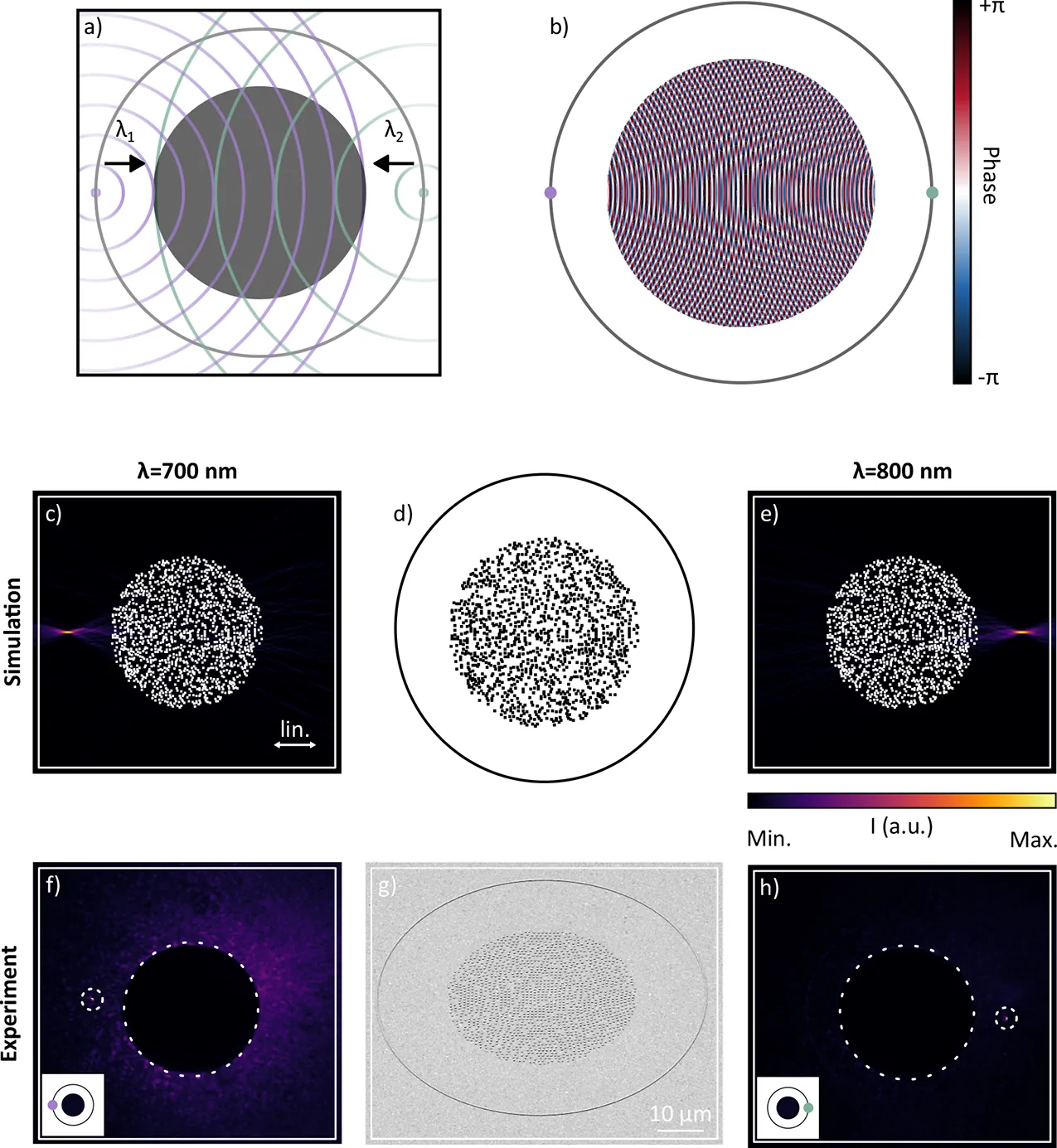
Design concept and near-field characterization of the two-wavelength device. (a) Two Huygens waves evolve from the backscattering element propagating toward the active area and form an interference pattern there. The phase profile of both interfered waves are shown in (b). (c and e) Constructive near-field interference under λ = 700 nm illumination produces a localized focal region on the left side of the coupling structure. Under 800 nm illumination, the interference of the plasmons creates a focal point on the right side. (d) To measure the plasmon intensity in the far-field, a scattering ring is integrated around the structure. (f and h) Respective experimental measurements of the two-wavelength device. (g) SEM image of the wavelength-demultiplexing metasurface.

Due to optical reciprocity, the efficiency of coupling free-space light to SPPs is inherently tied to their radiative out-coupling rate. Consequently, while high in-coupling efficiencies are beneficial, they also result in strong radiative damping and severely limit the SPP propagation lengths. Therefore, our design strategically targets the weak-scattering regime. By using shallow scatterers, the individual out-coupling cross-section remains low, minimizing radiative losses per scatter.^42,43^ This preserves the overall SPP propagation length and allows the spatial density of the scatterers to be maximized, ultimately providing significantly more degrees of freedom to precisely engineer the SPP interference field. The corresponding experimental results are depicted in Figure 3f and h. It should be noted that while the simulation depicts the entire near-field intensity distribution, the experimentally accessible measurement area is limited to the position of the backscattering ring. The expected position of the focal spot is illustrated in the CCD images on the bottom left in the schematic sample structure. As visible in the CCD images, high intensity is obtained at the focal spots, while the rest of the backscattering ring remains dark. All images are taken at 0.1 s integration time and are acquired at a maximum pixel intensity of 10 000 counts. Figure 3g displays an SEM image of the full structure of the calculated design in d. The active area has a diameter of 40 μm, while the backscattering ring is 70 μm in diameter. The lateral size of the metasurface is chosen such that the maximum distance between a scatterer and the ring remains within the propagation length, ensuring a sufficient signal amplitude.

This novel structural design enables effective control of surface waves and represents a unique approach that has not been widely reported in previous studies. The experiment effectively extracts locally the relevant amplitude information, proving the functionality as a compact wavelength demultiplexing device. As a next step, we extend this design concept to devices capable of operating at more than two wavelengths. Theoretical spectral limits are investigated and can be found in Figure S1. When going to more wavelength operation and keeping a centrosymmetric design, the focal spot distance should be maximized to ensure the best resolution. In order to launch SPPs efficiently in all in-plane directions, circularly polarized light is required for the demultiplexing of three and four wavelengths. The respective calculated designs are depicted in Figure 4 on the left. For all experimentally obtained images, the large dashed circle within the CCD image outlines the iFS aperture, which absorbs the specular reflection. The small dashed circles denote the respective focal spots. The first row depicts the experimentally obtained individual in-plane focal spots for the three-wavelength device. The images are taken under illumination with RCP light at 790, 850, and 900 nm, respectively. The second row presents the CCD images for the four-focal-spot device, obtained at 740, 800, 850, and 900 nm. Corresponding simulated field distributions can be found in Figure S2 of the supporting material. Distinct focal spots can be observed at the specified wavelength, with their expected positions additionally indicated in the schematic in the lower left panel. Moreover, the inverse design simulations exhibit also good agreement with the experimental results for the metasurfaces operating at three and four wavelengths. Comparing these two measurement sets with those of the two-foci device in Figure 3, it is clearly visible that more wavelengths increase the noise in the images. The explanation is 2-fold. Reducing the number of scatterers for each additional wavelength lowers the quality of the focal spot. On the other hand, the setup is aligned at wavelengths in the visible. Operating the setup and the device over a broader wavelength range introduces chromatic aberrations arising from propagation through multiple optical elements, including the objective, thereby increasing background noise and reducing imaging fidelity. The relative efficiencies of each device for the respective output channels are provided in Supplementary Table S2.

**Figure 4:**
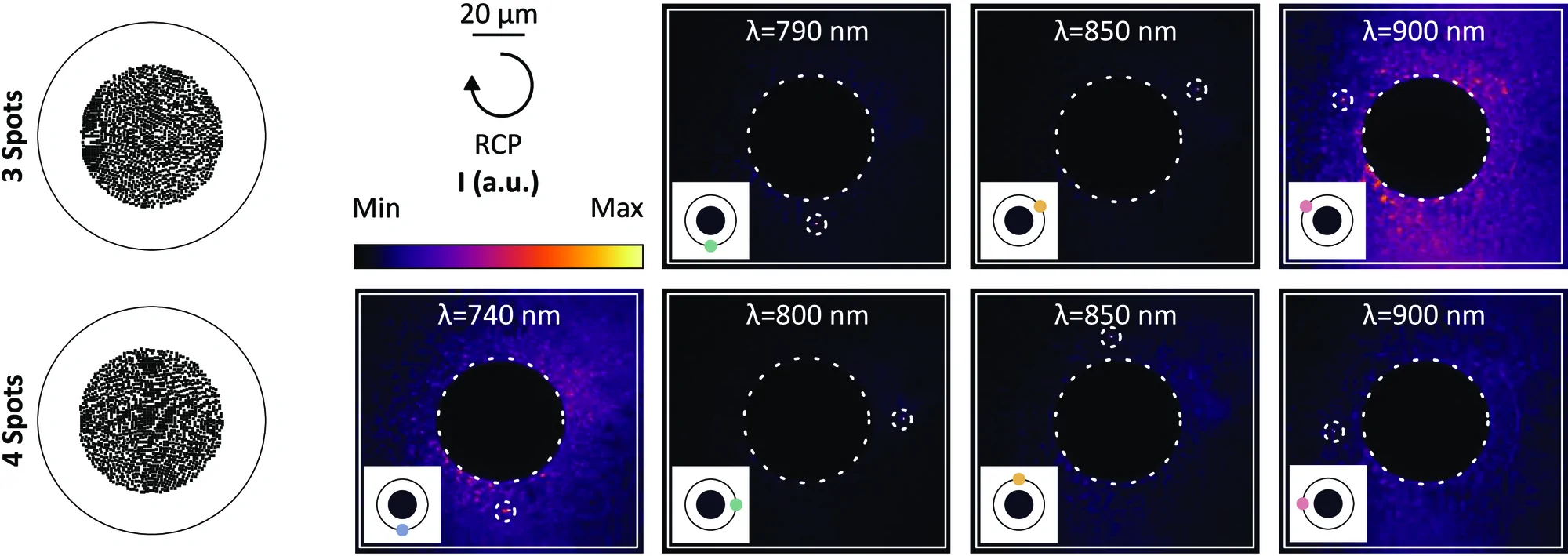
Multiwavelength measurements. Three- and four-wavelength demultiplexing devices, which are illuminated with one wavelength at a time under right handed circularly polarized light, create distinct focal spots. In the first column, the designs of both devices are shown together with the scattering ring. Individual scatterers have an edge length of 400 nm.

Multiwavelength demultiplexing of the metasurfaces is presented in Figure 5. Subpanels a-c depict the CCD images of simultaneous two, three, and four-wavelength demultiplexing. A zoom-in to the focal regions is shown in d-f, as well as the backscattering ring and the inverse field stop. Ten pixels of each focal spot are displayed to underline the focal character. The created focal spots in all devices are very narrow and cannot be experimentally resolved. Therefore, the radiative focal spot has to be imaged slightly above the sample plane which introduces defocusing along the *z*-axis and results in a broadened imaged focal width less than 4 μm. This corresponds to 10 pixels with a single pixel size of 433 nm × 433 nm. A detailed numerical analysis of the focal spot size is presented in Supplementary Section S3.5. The spectra of the focal regions are analyzed by integrating the signal over 5×5 pixel areas centered on each focal spot. Resulting spectra are subsequently normalized and plotted as a function of wavelength in Figure 5g–i. Dashed lines represent the simulated data, while the dots represent measurement points from the wavelength sweep. The images exhibit good agreement between simulation and measurements. Nevertheless, in the CCD images in Figure 5a-c, it is again demonstrated that with increasing wavelengths, the background noise in the images increases as well. Here, all wavelengths are visible simultaneously and highlighted with the small dashed circle. The schematic device on the bottom left in the CCD images highlight the spots of interest in the sample. The spectral behavior of the devices in Figure 5g-i show good overall agreement, but also for some cases a slight spectral shift. For instance the peak for λ = 850 nm in Figure 5h and is slightly red-shifted. For the 900 nm focal spot, it is only possible to investigate the behavior up to λ = 900 nm as this is the maximum wavelength of the laser tuning range.

**Figure 5:**
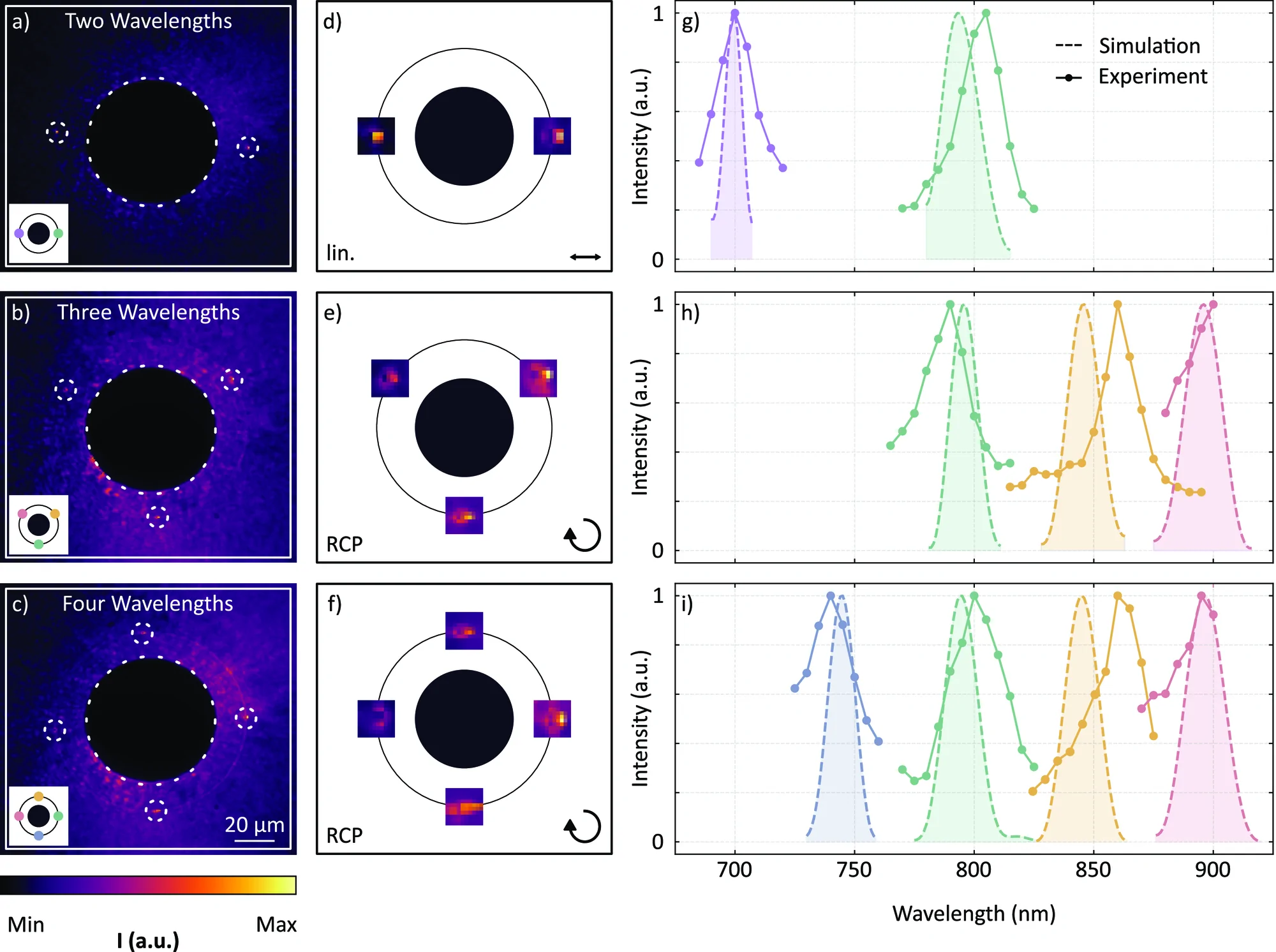
Near-field images and spectral response. (a–c) CCD images of the near-field distributions for configurations with 2, 3, and 4 focal spots, respectively. The corresponding excitation wavelengths are 700 and 800 nm (2 foci), 790, 850, and 900 nm (3 foci), and 740, 800, 850, and 900 nm (4 foci). In all cases, the operating wavelengths are simultaneously demultiplexed and spatially resolved under polarization indicated in (d)–(f). Here, zoom-ins of the individual obtained foci are illustrated. (g–i) Corresponding spectral response of the individual devices, quantified as the integrated intensity at the expected focal positions on the sample.

In conclusion, we have demonstrated plasmonic near-field wavelength demultiplexing for up to four wavelengths using engineered disordered design principles in combination with a template-stripping method for ultrasmooth gold surfaces. A full optical setup is built to locally access the constructively interfered plasmon waves. The presented approach could also enable demultiplexing and other functionalities based on attributes such as polarization, orbital angular momentum, or even vectorial properties of light. Beyond binary sample design, a multistep overlay etching process, focused ion beam milling, or thermal scanning-probe lithography could be utilized to gain control over the amplitude of the plasmon waves^15,34^ . This results in more degrees of freedom and might thus enable even more enhanced functionalities. By controlling the amplitude and phase of the SPPs at the focal points, this concept might find application in coherent photonic circuits. In combination with integrated near-field detectors^9,44^ , such architectures pave the way toward highly compact and scalable nanophotonic devices. The capability to create nearly arbitrary near-field distributions opens new opportunities for nanoscale optical routing, polarization-selective signal processing, and orbital-angular-momentum-dependent light manipulation.^42,45–51^ Moreover, such plasmonic metasurfaces, as well as analogous implementations in dielectric nanophotonic platforms may facilitate advanced coupling schemes to quantum emitters and other nanoscale systems, paving the way toward ultracompact integrated photonic receivers, multiplexers, and on-chip optical interconnects.^23,24,52,53^

## Supplementary Material



## Notes

General description of SPP launching at individual scatterers, criteria, and positions used for phase selection; design parameters of the scatterers including dimensions, density, and phase range, together with the associated symmetry considerations and the dependence on focal spot size and numerical aperture; detailed analysis of the spectral response, channel efficiency, and channel efficiency; spectral crosstalk analysis; and calculated field distributions for three- and four-wavelength devices (PDF)
