# Supplementary material for: Disordered Near-Field Metasurfaces for On-Chip Wavelength Demultiplexing
Source: Nano Lett. 2026 Oct 2;26(40):13955–61. doi: 10.1021/acs.nanolett.6c03564 (PMC13674331; doi:10.1021/acs.nanolett.6c03564)
Supplement: Supplementary file 1 [file nl6c03564_si_001.pdf]

Supporting Information for Disordered near-field metasurfaces for on-chip wavelength demultiplexing

L. Schmid*[1], J. Schwab[1], F. Kadriu[1], R. Horvat[1], C. Li[2], S. A. Maier[2,3], H. Giessen[1], H. Ren[2], and M. Hentschel[1]

[1] *4th Physics Institute and Research Center SCoPE, University of Stuttgart, Pfaffenwaldring 57, 70569 Stuttgart, Germany*
[2] *School of Physics and Astronomy, Monash University, Clayton, Victoria, Australia*
[3] *Department of Physics, Imperial College London, London, SW7 2AZ, United Kingdom*

*Email: luca.schmid@pi4.uni-stuttgart.de

Contents

S1 Simulation Methods 2

S2 Inverse Design Methodoloy 2

S3 Analysis of Design Parameters 3
- S3.1 Scatterer dimensions . . . 3
- S3.2 Scatterer density . . . 3
- S3.3 Phase range . . . 5
- S3.4 Symmetry considerations . . . 5
- S3.5 Focal Spot Size and NA Dependence . . . 5

S4 Spectral Response and Channel Efficiency 7

S5 Crosstalk Analysis 9

S6 Calculated Near-field distributions 10

S1 Simulation Methods

To model the near-field metasurface, individual scatterers are treated as surface plasmon polariton point sources. The electric field $\boldsymbol{E}_\mathrm{s}(\boldsymbol{r},t)$ at position $\boldsymbol{r}$ and time t, launched by a single scatterer, is expressed as

$$\boldsymbol{E}_\mathrm{s}(\boldsymbol{r},t) = E_0\ e^{-i\omega_0 t}\ e^{-k_z z}\left(\widehat{\boldsymbol{e}}\cdot\widehat{\boldsymbol{r}}_\mathrm{s}\right)\left(\frac{ik_z\widehat{\boldsymbol{r}}_\mathrm{s} - k_\mathrm{p}\widehat{\boldsymbol{z}}}{k_0}\right)H_0^{(1)}\left(k_\mathrm{p}r_\mathrm{s}\right), \tag{S1}$$

where k_p and k_z represent the complex-valued in-plane and out-of-plane components of the SPP wave number, respectively. These are derived from the vacuum wave number k_0 using the relations $k_\mathrm{p} = k_0\sqrt{\frac{\epsilon_\mathrm{m}\epsilon_0}{\epsilon_\mathrm{m}+\epsilon_0}}$ and $k_z^2 = k_\mathrm{p}^2 - k_0^2$ at the bulk gold-air interface. The permittivity of gold, ϵ_m, is obtained from tabulated values [1]. For example, at an operating wavelength of $\lambda = 2\pi/k_0 = 800\,\mathrm{nm}$, the relative permittivity is approximately $\epsilon_\mathrm{m}/\epsilon_0 = -22.78+0.76i$, yielding an in-plane wave number of $k_p = (8.03+0.0062i)\,\mathrm{\mu m}^{-1}$. Additionally, ω_0 denotes the angular frequency, $\widehat{\boldsymbol{e}}$ is the polarization unit vector of the incident light, with $\widehat{\boldsymbol{e}} = 1/\sqrt{2}(1,+\mathrm{i})$ for LCP light and $\widehat{\boldsymbol{e}} = 1/\sqrt{2}(1,-\,\mathrm{i})$ for RCP light and $H_0^{(1)}(k_\mathrm{p}r_\mathrm{s})$ is the zeroth-order Hankel function of the first kind. The distance vector from the source at $\boldsymbol{r}'$ to the observation point $\boldsymbol{r}$ is defined as $\boldsymbol{r}_\mathrm{s} = \boldsymbol{r}-\boldsymbol{r}'$, with its corresponding unit vector $\widehat{\boldsymbol{r}}_\mathrm{s} = \boldsymbol{r}_\mathrm{s}/r_\mathrm{s}$. The total electric field generated by the entire metasurface is calculated as the coherent superposition of Huygens wavelets emitted by all scatterers. This linear summation neglects multiple scattering events and near-field mode coupling between adjacent structures. As discussed in the main text and section S3, this weak-scattering approximation is justified by the shallow geometry of the individual scatterers, which minimizes their radiative out-coupling cross-section.

S2 Inverse Design Methodoloy

The spatial positions of the individual scatterers across the near-field metasurface is determined using a time-reversed inverse design approach. Instead of simulating the forward SPP propagation of scattered light, the algorithm calculates the optimal scattering coordinates by simulating the backward propagation of SPPs from the focal positions to the near-field metasurface. First, cylindrical Huygens waves are launched at the intended focal points located at the backscattering ring. These waves also follow Equation S1, where the target wavelength and polarization state determine their excitation amplitude and phase in Equation S1. The interference of these backward-propagating SPP waves in the central metasurface region is calculated with a resolution of 100 nm so that it can be used as a starting point for the scatterer placement. Subsequently, scatterers are assigned to spatial locations exhibiting a specific constructive phase range

$$\phi_0 - \phi_\mathrm{r} \leq \phi \leq \phi_0 + \phi_\mathrm{r}. \tag{S2}$$

This ensures that plasmons launched from these specific coordinates will interfere constructively at the target focal points in the forward-propagating scenario. A quantitative comparison of different phase ranges ϕ_r is presented in section S3. Finally, the discrete coordinates extracted from the field maps are stochastically populated with scatterers. During this assignment step, a spatial exclusion radius (dictated by the physical dimensions of the scatterers) is used to prevent physical overlap of adjacent scatterers.

S3 Analysis of Design Parameters

The design methodology introduced in section S2 depends on a few parameters. Their effects of on the resulting performance is subsequently discussed.

S3.1 Scatterer dimensions

The physical dimensions of the scatterers determine the coupling efficiency of incident light to SPP modes, which is inherently connected to the out-coupling efficiency of SPP modes. Our template-stripping fabrication method dictates specific geometric constraints, yielding protruding shallow gold islands rather than deep trenches. While previous characterizations of similar nanostructures by Spektor et al.[2] indicate that the coupling efficiency reaches a theoretical maximum at a scatterer width of 400 nm, the overall coupling strength in our system remains exceptionally low because the island height is restricted by the 50 nm dry-etching depth, because of the subsequent stripoff process. Consequently, this shallow height profile ensures that the metasurface operates strictly within the weak-scattering regime, which advantageously minimizes radiative losses per individual scatterer and preserves the overall SPP propagation.

S3.2 Scatterer density

With the dimensions of the scatterers being fixed to a width of 400 nm and a height of 50 nm, the total fields can be estimated by a linear summation of the fields of individual scatterers (see section S1). As a result, the total calculated intensity in the focal spots is expected to scale approximately linearly with the number of scatterers. In experiments, however, placing scatterers too densely can introduce issues. Since these issues depend on specific experimental techniques, we evaluate the underlying concept using idealized simulations that assume no such constraints. This applies to Figure S1, S2, and S4. For these specific cases, we maximize the scatterer density by placing as many scatterers as possible that fulfill Equation S2 without physically overlapping. Conversely, in simulations intended to reproduce our experimental devices, we model the exact realized samples, which have a reduced amount of scatterers. This applies to all simulations presented in the manuscript, and Figure S5. The number of scatterers of all designs presented in the manuscript and the supplement are listed in Table S1.

Design	Amount of Scatterers
two-wavelength demultiplexing	1260
three-wavelength demultiplexing	1349
four-wavelength demultiplexing	1398
$\phi_\mathrm{r} = 0.05\pi$	2271
$\phi_\mathrm{r} = 0.1\pi$	2689
$\phi_\mathrm{r} = 0.2\pi$	2924
$\phi_\mathrm{r} = 0.4\pi$	3064
$\phi_\mathrm{r} = 0.6\pi$	3478
$\phi_\mathrm{r} = 0.8\pi$	4386
$\phi_\mathrm{r} = \pi$	4649

Table S1: Amount of scatterers for all experimentally realized metasurfaces presented in the main text and the test metasurface designs of Figure S1.

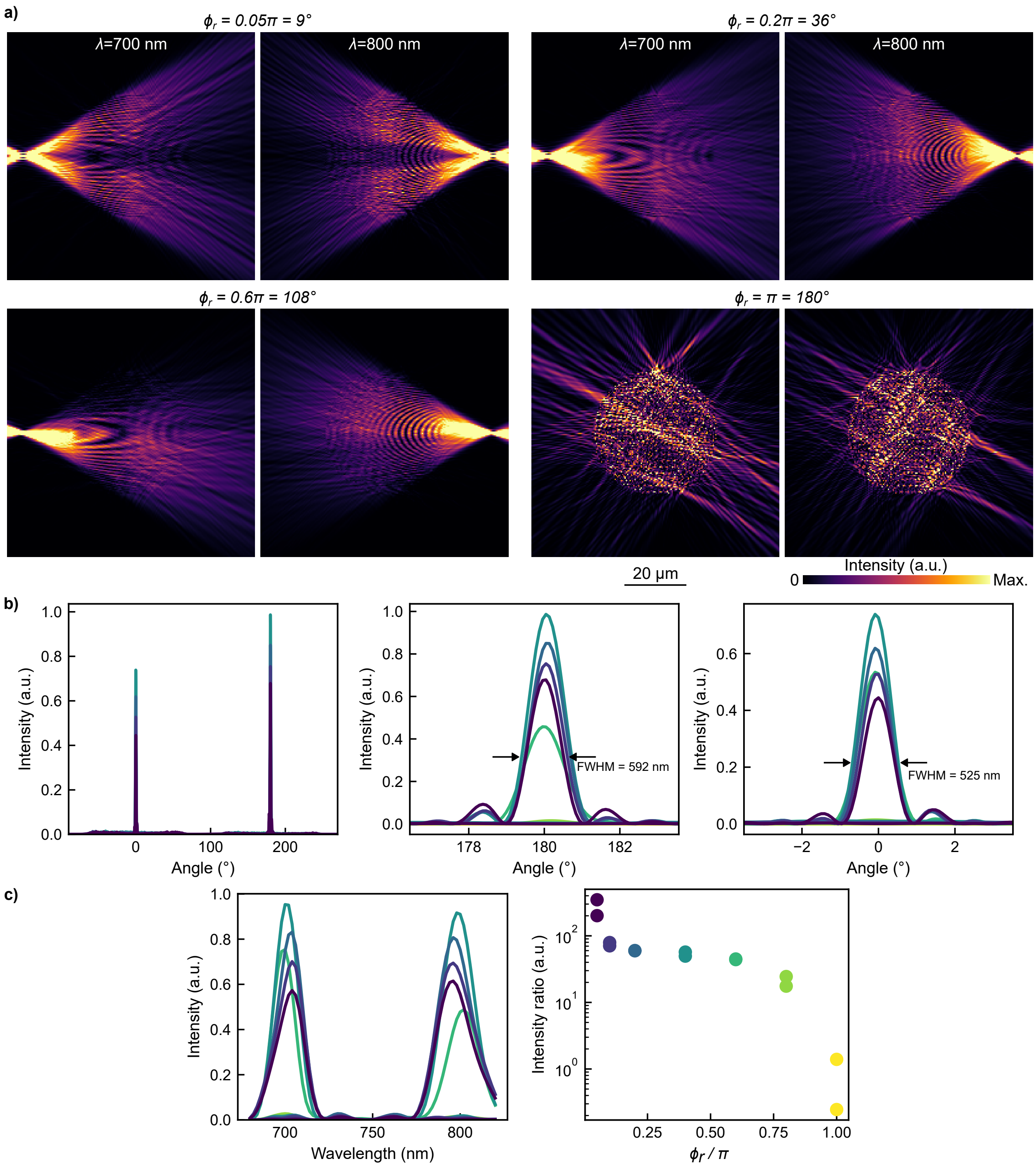

Figure S1: **Comparison of the two-wavelength-demultiplexing meatasurface performance for different values of ϕ_r.** **a**, Intensity distributions at excitation wavelengths of $\lambda = 700\,\text{nm}$ and $800\,\text{nm}$. **b**, Intensity distribution along the backscattering (radius of $35\,\mu\text{m}$), with a zoom-in on the focal points. The colors correspond to different values of ϕ_r and can be read out in the right plot of panel c. The two curves at each value of ϕ_r correspond to $\lambda = 700\,\text{nm}$ and $800\,\text{nm}$, respectively. **c**, Integrated intensities of the focal points as a function of the illumination wavelength. In the right panel, the ratio between the intensity at the target wavelength and the intensity at the corresponding other wavelength is depicted for each focal position.

S3.3 Phase range

With the scatterer dimensions being fixed and the scatterer density maximized and tied to Equation S2 (for the following concept study, see subsection S3.2), the parameter ϕ_r in Equation S2 is the only free parameter in the design methodology of section S2. In the following, we analyze its impact on the resulting performance of the near-field metasurface based on the results presented in Figure S1. From the intensity distributions depicted in panels a and b, it becomes apparent that smaller values of ϕ_r and therefore a more stricter phase mask, result in a higher demultiplexing performance, while sacrificing efficiency. The lower efficiencies for lower values of ϕ_r stem from the lower number of pixels fulfilling Equation S2 and the resulting lower number of scatterers in the final design. The number of scatterers for all designs are listed in Table S1.

S3.4 Symmetry considerations

The near-field metasurface for separating two wavelengths (presented in Fig. 3 of the main text) exhibits a mirror-symmetry along the y-axis. This symmetry is not considered in the design methodology of section S2. In the following, we present the results of a related alternative design method, which takes the symmetry into account. In this approach, the interference field of the back-propagated waves is calculated only for the upper half of the metasurface area with $y \geq 0$. For every scatterer placed in this domain, another scatterer will be placed in the lower mirror-symmetric domain $y < 0$. The performance of both methods are compared in Figure S2. As expected, the mirror-symmetric designs results in mirror-symmetric intensity distributions and symmetric focal points. However, the spectral demultiplexing performance remains comparable.

S3.5 Focal Spot Size and NA Dependence

Figure S1 and S2 present the numerically obtained focal spots, where different values of the phase range ϕ_r result in similar spatial confinement. For all plotted parameters, the focal spots their measured FWHM is below or close to the SPP wavelength. The minimum FWHM of each focal spot is denoted in the respective figures, demonstrating sub-wavelength confinement for both $\lambda_\mathrm{SPP} = 677\,\mathrm{nm}$ ($\lambda = 700\,\mathrm{nm}$) and $\lambda_\mathrm{SPP} = 782\,\mathrm{nm}$ ($\lambda = 800\,\mathrm{nm}$). The NA of the metasurfaces is governed by the radius of the metasurface relative to the size of the backscattering ring. It is calculated here as $\mathrm{NA} \approx \sin(\arctan(20/35)) \approx 0.5$, where $20\,\mathrm{\mu m}$ is the radius of the metasurface and $35\,\mathrm{\mu m}$ is the radius of the backscattering ring. Lower NAs can be realized by reducing the metasurface radius and increasing the backscattering ring radius, while higher NAs are achieved by shrinking the radial distance to the backscattering ring relative to the metasurface radius.

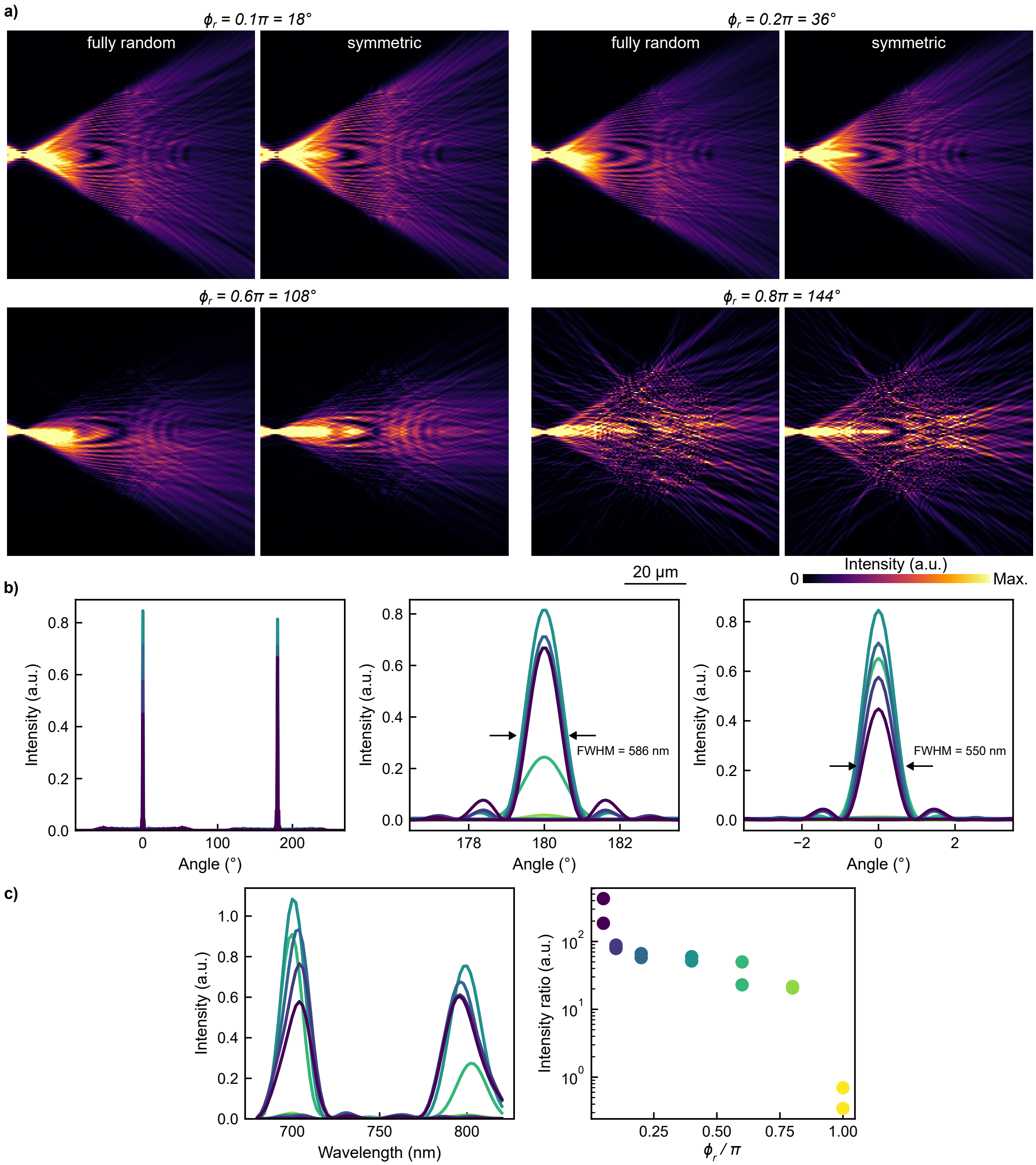

Figure S2: **Comparison of the design methodology with a mirror-symmetric metasurface design approach.** **a**, Intensity distributions at an excitation wavelength of $\lambda = 700\,\text{nm}$ for the fully random and the mirror-symmetric design. **b**, Intensity distribution along the backscattering (radius of 35 µm) of the mirror-symmetric design, with a zoom-in on the focal points. The colors correspond to different values of ϕ_r and can be read out in the right plot of panel c. The two curves at each value of ϕ_r correspond to $\lambda = 700\,\text{nm}$ and 800 nm, respectively. **c**, Integrated intensities of the focal points as a function of the illumination wavelength of the mirror-symmetric design. In the right panel, the ratio between the intensity at the target wavelength and the intensity at the corresponding other wavelength is depicted for each focal position.

S4 Spectral Response and Channel Efficiency

Fully obtained spectral response and the corresponding relative efficiency is illustrated for the targeted wavelengths calculated as $\eta_{\mathrm{rel},i} = \frac{I_i}{\sum_j I_j}$, where I_i denotes the integrated counts at the targeted focal position and the sum runs over all measured focal positions.

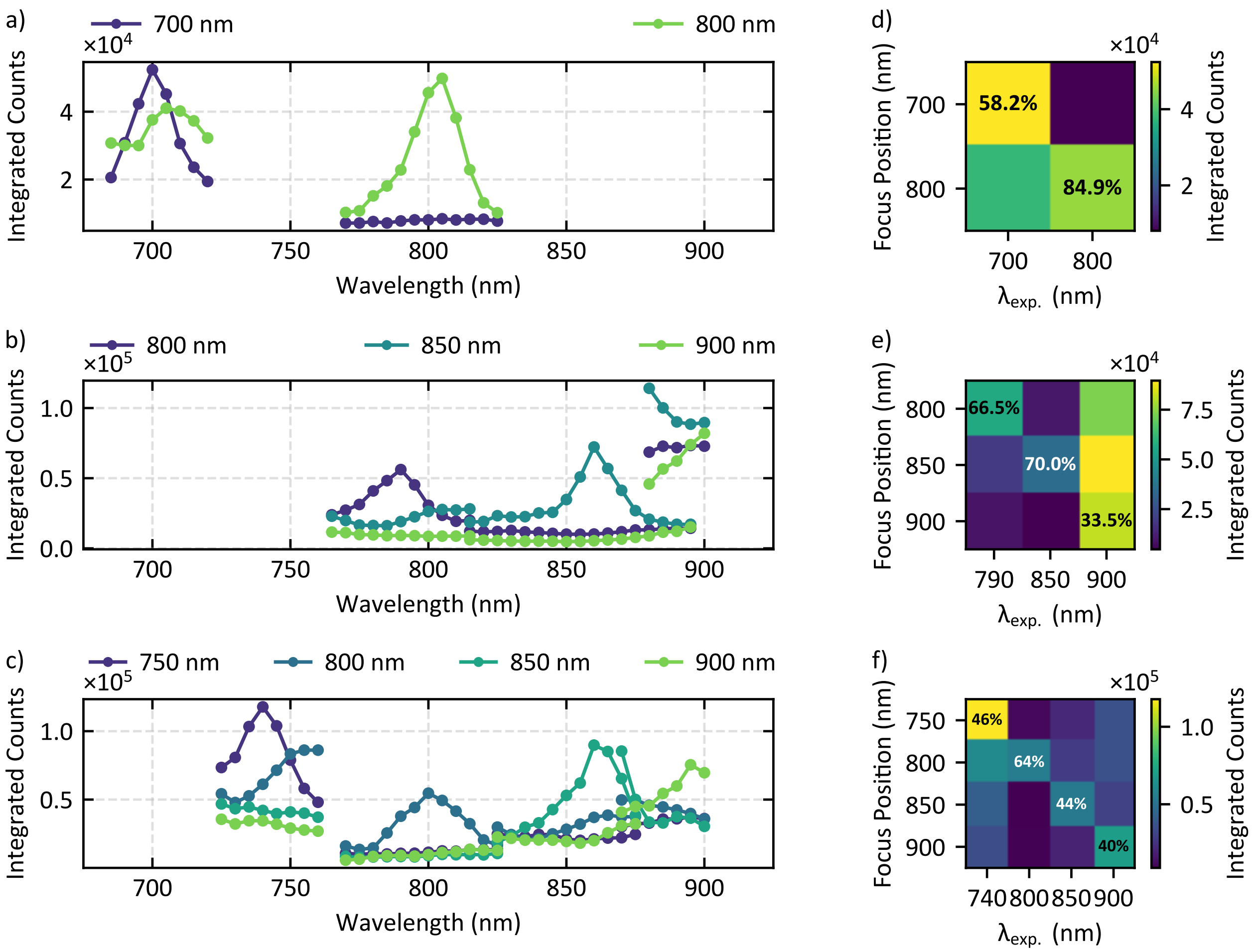

Figure S3: **Spectral response and relative efficiency. a-c**, Full spectral response data obtained for the three devices. Integrated counts at the targeted focal position are shown as a function of the probed wavelength, together with the integrated counts from the non-targeted focal positions. **d-f** Relative efficiency matrices color-coded for all three devices with the relative efficiency on the diagonal. The y-axis denotes the probed focal position, while the x-axis indicates the corresponding excitation wavelength. For the three-wavelength device, the relative efficiency at 900 nm is reduced to 33.5 %. This reduced efficiency is attributed to the increased background noise observed across the corresponding measurement, as shown in Figure 4 which contributes to the integrated signal at the non-targeted focal positions. Similar crosstalk is visible for the four-wavelength device at 740 nm.

Focus Positions (nm)	$\lambda_{exp.}$ (nm)	
	700	800
700	58.2 %	15.1 %
800	41.8 %	84.9 %

Focus Positions (nm)	$\lambda_{exp.}$ (nm)		
	790	850	900
800	66.5 %	20.4 %	29.8 %
850	22.6 %	70.0 %	36.7 %
900	10.9 %	9.6 %	33.5 %

Focus Positions (nm)	$\lambda_{exp.}$ (nm)			
	740	800	850	900
750	46.1 %	13.5 %	17.4 %	21.0 %
800	24.0 %	64.4 %	23.2 %	20.8 %
850	16.5 %	10.6 %	43.5 %	17.7 %
900	13.5 %	11.5 %	15.9 %	40.5 %

Table S2: Relative efficiencies of the three devices as a function of focus position and excitation wavelength.

S5 Crosstalk Analysis

Simulated crosstalk for wavelength demultiplexing with varying spectral distance $\Delta\lambda$ is presented in Figure S4.

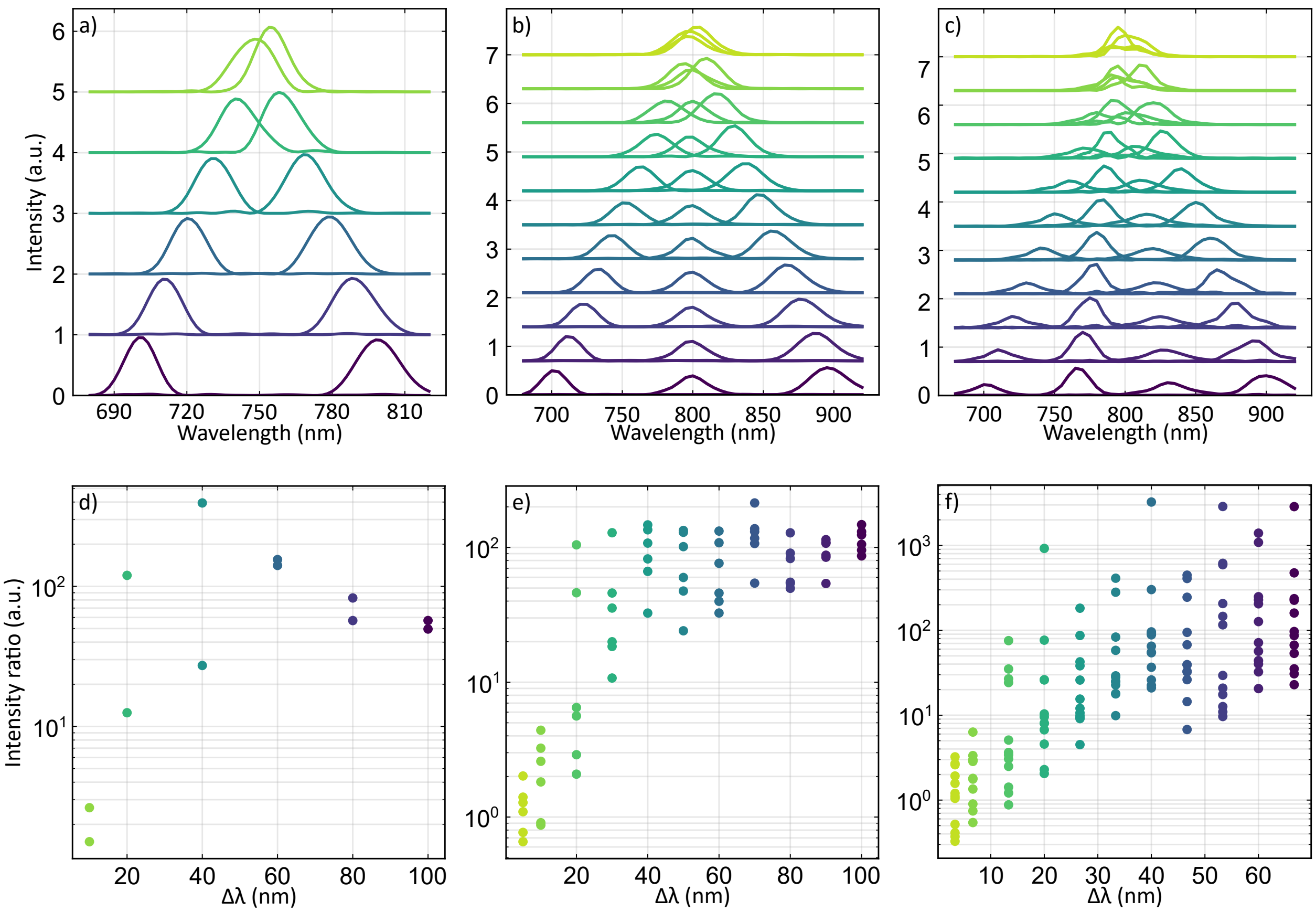

Figure S4: **Spectral limits of near-field metasurfaces. a-c** Waterfall plots illustrating the spectral behavior of the devices separating two, three, and four wavelengths. The colors indicate distinct near-field metasurfaces with decreasing spectral distance of the separated wavelengths. **d–f** Ratio of the intensity of the intended focal spots over the intensity at each other focal position. These intensity ratios are calculated at each design wavelength of the devices.

S6 Calculated Near-field distributions

Calculated near-field distributions for three- and four-wavelength demultiplexing are shown, illustrating the spatial field distributions and wavelength-dependent routing at the corresponding output channels.

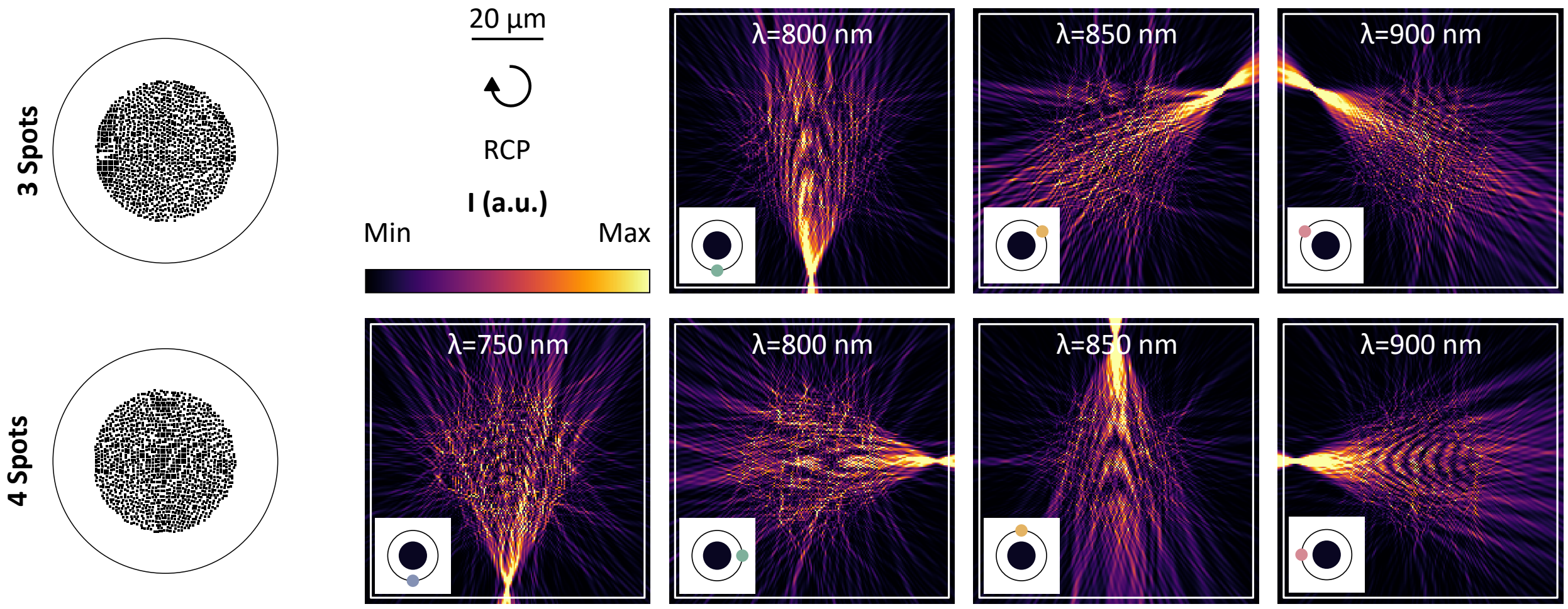

Figure S5: **Multi-wavelength calculated field distributions.** Three- and four-wavelength demultiplexing devices with only one illuminating wavelength at once, demonstrating the spatial separation into individual focal spots. In the first column, the designs of both devices are depicted with the scattering ring. Individual scatterers have an edge length of 400 nm.

References

(1) Weaver, J.; Frederikse, H., *CRC Handbook of Chemistry and Physics*; CRC Press: 2014.

(2) Spektor, G.; Prinz, E.; Hartelt, M.; Mahro, A.-K.; Aeschlimann, M.; Orenstein, M. Orbital angular momentum multiplication in plasmonic vortex cavities. *Sci. Adv.* **2021**, *7*, eabg5571.
